# In-Hospital Glycemic Variability and Mean Blood Glucose as Risk Markers for Long-Term Mortality in Patients with Diabetes

**DOI:** 10.3390/jcm14248820

**Published:** 2025-12-12

**Authors:** Mónica Sachi Martínez-Mihara, Pablo Lozano-Martínez, Ana Belén Mañas-Martínez, José Antonio Gimeno-Orna, Daniel Sáenz-Abad

**Affiliations:** 1 Department of Emergency Medicine, Lozano Blesa University Hospital, 50009 Zaragoza, Spain; monicasachi@hotmail.com; 2 Aragon Health Research Institute (IIS Aragon), 50009 Zaragoza, Spain; pablolozano7@gmail.com (P.L.-M.); abmannas@salud.aragon.es (A.B.M.-M.); jagimeno@salud.aragon.es (J.A.G.-O.); 3 Department of Medicine, Psychiatry and Dermatology, Faculty of Medicine, University of Zaragoza, 50009 Zaragoza, Spain; 4 Department of Endocrinology and Nutrition, Lozano Blesa University Hospital, 50009 Zaragoza, Spain

**Keywords:** mean glucose, glycemic variability, coefficient of variation, mortality

## Abstract

**Background/Objectives**: This study aimed to evaluate the influence of mean blood glucose (MG) and glycemic variability (GV) during hospitalization on the risk of mortality in patients with diabetes mellitus (DM). **Methods**: We conducted a retrospective cohort study including patients with DM admitted to the Internal Medicine ward. The dependent variable was post-discharge mortality. Capillary glucose levels were collected, and MG, standard deviation (SD), and coefficient of variation (CV = SD/MG) were calculated. The predictive value of MG and GV, expressed as CV, for mortality was assessed, adjusting for hypoglycemic episodes and comorbidities. Survival analysis and multivariate Cox regression were performed. **Results**: A total of 276 patients were included, mean age of 77.6 ± 10.2 years, 146 (52.9%) were males. Heart failure was the leading cause of admission (40.4%). During a median follow-up of 2.7 years, 249 patients (90.2%) died, corresponding to 212 deaths per 1000 patient years (289 with CV > 0.29 vs. 168 with CV ≤ 0.29). In the multivariate Cox model, CV > 0.29 (HR = 1.60; 95% CI 1.23–2.08; *p* = 0.001) and MG > 140 mg/dL (HR = 1.71; 95% CI 1.16–2.51; *p* = 0.004) were independent predictors of mortality. **Conclusions**: Elevated MG and GV measured during hospitalization may help stratify mortality risk after discharge in patients with DM

## 1. Introduction

Diabetes mellitus (DM) is a chronic disease with a high prevalence (10.3%) among adults aged 20 to 79 in Spain [1]. Furthermore, the Global Burden of Disease [2] projection in Spain for 2050 estimates that prevalence could increase by more than 30%. Due to its significant impact on patients’ lives and at a socioeconomic level, this condition represents a global public health challenge: it is one of the four chronic diseases responsible for 70% of total global mortality, alongside cardiovascular disease, cancer, and respiratory disease [3].

In fact, an analysis of data from the cohorts included in the Emerging Risk Factors Collaboration establishes that the presence of DM doubles the risk of all-cause and cardiovascular mortality [4], with the effect being more pronounced the earlier the onset of the disease [5]. Therefore, it is crucial to stratify patients’ mortality risk and determine the influence of metabolic control of the disease on that risk [6]. Attention must be paid not only to mean glucose concentrations (MG), as reflected in HbA1c values, but also to glycemic variability (GV) [7].

In recent years, attention has been directed toward the importance of glycemic variability as a relevant parameter in the metabolic control of DM [8]. GV can be measured in the short-term by using the coefficient of variation (CV) of daily glucose values, or in the long-term by using the CV of HbA1c values across successive visits [9].

However, the independent and additive contribution of GV to the well-established harmful effects of chronic hyperglycemia on the risk of complications remain controversial [10]. The mechanism by which GV may increase the risk of complications is thought to involve a higher likelihood of hypoglycemic episodes, as well as its associations with inflammation and oxidative stress at the endothelial level [11,12].

In hospitalized patients, hyperglycemia, hypoglycemia, and GV have all been associated with increased morbidity and mortality, highlighting the need to implement effective management protocols. These should include capillary blood glucose monitoring and insulin regimens that address basal and prandial needs, as well as provide corrective doses when necessary [13].

Studies have suggested that elevated GV during hospitalization may be associated with increased mortality after hospital discharge [14,15], although often without a proper adjustment for MG values. Our group has previously confirmed that GV in patients with diabetes during hospitalization is associated with increased mortality, both during hospitalization [16] and in the short-term follow-up [17]. Determining whether this trend persists over a longer follow-up period could provide valuable information to improve treatment.

Our working hypothesis is that both MG and GV during a hospital stay may act as predictive, additive, and independent factors for long-term mortality in patients with DM.

## 2. Materials and Methods

### 2.1. Design

Retrospective, analytical, and longitudinal cohort study.

### 2.2. Patients

A more detailed description of the cohort can be found in previous publications [16,17]. The cohort included patients discharged from the Internal Medicine ward of the Hospital Clínico Universitario Lozano Blesa of Zaragoza with a diagnosis related to DM during two periods: from January to April 2010 and from January to April 2013. Patients whose primary diagnosis were diabetic ketoacidosis or hyperosmolar hyperglycemic non-ketotic state, those who died during hospitalization, and those who were admitted to Intensive Care Unit (ICU) were excluded. The original study [17] was designed to have an 80% power to detect a minimum mortality difference of 15%.

### 2.3. Variables

The principal dependent variable (clinical endpoint) was all-cause mortality after hospital discharge. Patients were followed from hospital discharge until death or until 1 August 2023, extending the follow-up period compared to our previous study [17] by 4 years.

The independent variables collected were:

- Clinical variables: age, gender, reason for admission, history of cardiac, respiratory, neurological, digestive, or neoplastic diseases, Charlson Comorbidity Index (CCI), blood pressure, oxygen saturation, temperature, hospital length of stay and type of DM and treatment received.
- Glycemic variables: capillary blood glucose levels during the hospital stay and the frequency and severity of hypoglycemia (defined as blood glucose < 70 mg/dL, considering severe hypoglycemia a blood glucose < 40 mg/dL or those causing loss of consciousness).
- Biochemical variables: plasma glucose, creatinine, electrolytes, HbA1c, and hemogram.
- Generated variables derived from capillary blood glucose values included: The following glycemic control measures were derived for each patient: maximum glucose level (the highest capillary blood glucose value determined during the hospital stay), the MG (the average of all capillary blood glucose levels), and GV estimated using the CV, defined as the ratio of the standard deviation (SD) of the capillary blood glucose values and the MG. Patients were stratified according to MG (with a cutoff at 140 mg/dL based on its clinical significance) and according to CV (with a cutoff at 0.29, which was the median of the distribution).

### 2.4. Laboratory Methods

Venous plasma glucose was measured using a hexokinase enzymatic method (Roche Diagnostics, Mannheim, Germany), while capillary glucose was obtained with the Optium Xceed® meter (Abbott Diabetes Care, Alameda, CA, USA), and HbA1c was determined by high-resolution chromatography (Bio-Rad Laboratories, Hercules, CA, USA). The remaining biochemical parameters and the complete blood count were analyzed using routine methods on an autoanalyzer. Glomerular filtration rate (GFR) was calculated using the CKD-EPI formula, with stratification according to the KDIGO classification [18].

### 2.5. Statistical Methods

Quantitative variables are described with their mean and standard deviation (±SD). Qualitative variables are described with their frequency distribution. Comparisons of quantitative variables were performed using Student’s *t*-test for independent samples of two groups and using ANOVA for samples of more than two groups, or with non-parametric tests such as Mann–Whitney (for two groups) or Kruskal–Wallis (for more than two groups). Comparison of qualitative variables was performed using the Chi-square test or Fisher’s exact test, with linear trend tests for variables with ordinal categories. Mortality rates per 1000 patient years were calculated, and comparison of rates was made using Kaplan–Meier survival curves, assessing statistical significance with the log-rank test. A Spearman correlation analysis was performed between glycemic variables to detect collinearity before their introduction into the regression models. Predictive factors for mortality were assessed using univariate and multivariable Cox regression analyses. Variables were considered for inclusion in the multivariate model based on statistical criteria (*p* ≤ 0.10 in the univariate analysis) and clinical relevance. To identify the best predictive model, we implemented a sequential backward elimination procedure in which variables were removed stepwise according to their relative predictive contribution. Specifically, the variables entered in the initial model were: age, sex, heart failure (HF), neurological disease, CCI, systolic blood pressure, oxygen saturation, estimated GFR, maximum glucose, MG, CV, presence and number of hypoglycemic episodes, and HbA1c. Model comparisons were performed using the likelihood-ratio test, and the final model included those variables that retained statistical significance while maximizing predictive performance. Assumptions of linearity and proportional hazards were verified through inspection of residual plots to confirm random distribution around zero and visual assessment of survival curves. Associations with *p* < 0.05 were considered statistically significant. Analyses were conducted using SPSS version 22.0 and R version 3.1.2.

## 3. Results

### 3.1. Descriptive Statistics

A total of 276 patients were included, with a mean age of 77.6 ± 10.2 years, of whom 146 (52.9%) were male. The most common reason for admission was dyspnea, with a diagnosis of HF in 111 patients (40.4%). Most patients (256) (92.8%) had type 2 DM, while 14 (5.1%) were diagnosed with stress hyperglycemia and 6 (2.2%) with type 1 DM or secondary diabetes. Patients presented with a high comorbidity burden at admission, with a history of cardiac disease in 152 (55.1%), neurological disease in 79 (28.6%), respiratory disease in 67 (24.3%), gastrointestinal disease in 42 (15.2%), and neoplastic disease in 37 (13.4%). The mean CCI score was 2.8 ± 1.6 points, with 47.1% of patients scoring < 3 points, 25.7% scoring 3 points, and 27.2% scoring > 3 points. The mean length of hospital stay was 13.9 ± 18.4 days.

The initial venous plasma glucose level was 194 ± 95 mg/dL. The MG level during hospitalization was 181 ± 41.7 mg/dL, the maximum glucose level was 300 ± 83.4 mg/dL, the SD was 55 ± 24 mg/dL, and the CV was 0.3 ± 0.1. The correlation between MG and CV was statistically significant but weak (R = 0.235; *p* < 0.001). The percentage of patients with MG > 140 mg/dL was 84.4% and 46.9% had a CV > 0.29. The mean number of hypoglycemic episodes was 0.66 ± 1.5 per patient. A total of 26.8% of patients experienced hypoglycemia during hospitalization: 13.4% had a single episode and 13.4% had multiple episodes, whereas 73.2% had no hypoglycemic events. There were 12 patients (4.4%) with severe hypoglycemia.

### 3.2. Comparison of Patients According to Their Characteristics

The mean follow-up duration was 4.3 ± 4.1 years, with a median of 2.7 years. After discharge, 226 patients (81.9%) required readmission during the follow-up period, of whom 56 (24.8%) were due to HF and 28 (10.1%) due to hypoglycemia. There were 249 deaths (90.2%), with only 27 patients (9.8%) alive as of 1 August 2023. Table 1 shows the general characteristics of the patients, and presents glycemic control measures depending on whether or not death occurred during the follow-up period. Non-survivors were older, had lower eGFR, higher CCI scores and a higher frequency of admissions due to HF. Regarding glycemic control, patients who died more frequently had a MG > 140 mg/dL and a CV > 0.29, as well as a higher mean number of hypoglycemic episodes.

Table 1 presents the univariate analysis of factors associated with mortality during follow-up. The table includes general clinical variables, comorbidities, renal function categories, vital signs, glycemic parameters on admission and during hospitalization, as well as hypoglycemia metrics. Hazard ratios (HR) with 95% confidence intervals (CI) and corresponding *p*-values are shown for each variable. HF: heart failure; SBP: Systolic Blood Pressure; GFR: Glomerular Filtration Rate; SD: Standard Deviation; CV: Coefficient of Variation.

Table 2 summarizes the general characteristics and glycemic variables of the patients according to their CV (≤0.29 or >0.29) and Table 3 does so according to their MG (≤140 or >140 mg/dL). Notably, both MG > 140 mg/dL and CV > 0.29 were associated with higher CCI scores, and patients with CV > 0.29 had a higher proportion of both total and severe hypoglycemia episodes.

Table 2 compares the baseline demographic, clinical, renal, and glycemic variables between patients with a glucose coefficient of variation (CV) ≤ 0.29 and those with CV > 0.29. Differences between groups are shown with their corresponding *p*-values. SBP: Systolic Blood Pressure; GFR: Glomerular Filtration Rate.

Table 3 summarizes the demographic, clinical, renal, inflammatory and glycemic characteristics stratified by mean glucose (MG) levels during hospitalization (≤140 mg/dL vs. >140 mg/dL). Group comparisons are reported with *p*-values. HF: heart failure; SBP: Systolic Blood Pressure; GFR: Glomerular Filtration Rate; SD: Standard Deviation; CV: Coefficient of Variation.

### 3.3. Mortality Rates

The overall mortality rate in the study population was 211.8/1000 patient years, being higher in men (222.7/1000) than in women (202.9/1000), although the difference was not statistically significant. Rates were significantly higher in patients with a MG > 140 mg/dL compared to those with MG ≤ 140 mg/dL (234.1/1000 vs. 137.4/1000; *p* = 0.006) and in patients with a CV > 0.29 compared to those with CV ≤ 0.29 (288.6/1000 vs. 168.2/1000; *p* < 0.001) (Table 4). Similarly, mortality rates exceeded 300 per 1000 patient years among those with a history of neurological disease (306.6/1000), a CCI score > 3 (326.7/1000), and an eGFR between 30 and 44 mL/min/1.73 m^2^ (310.6/1000).

Table 4 displays crude mortality rates per 1000 patient years across different subgroups, including sex, mean glucose categories, glucose variability categories, comorbidity burden (Charlson Comorbidity Index), renal function strata, and neurological disease status. For each subgroup, total follow-up time, number of deaths, and calculated mortality rates are presented. CCI: Charleston Comorbidity Index; CV: Coefficient of Variation; GFR: Glomerular Filtration Rate.

### 3.4. Predictive Factors of Mortality: Multivariate Analysis

**Figure 1:**
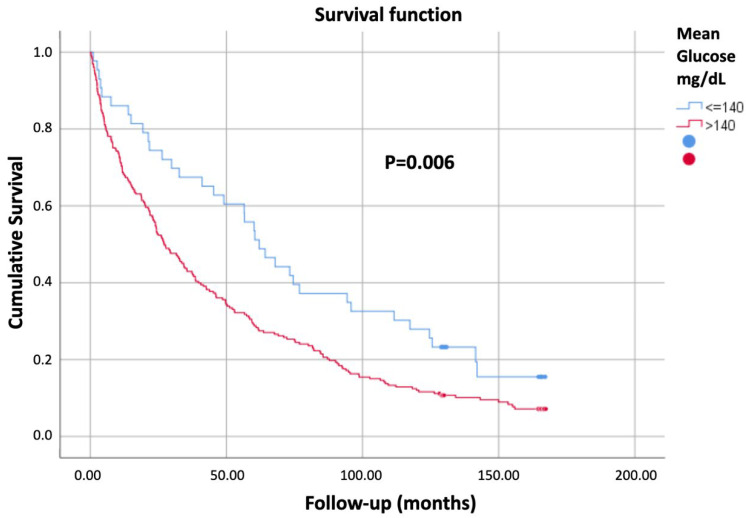
Survival curves by mean glucose levels.

**Figure 2:**
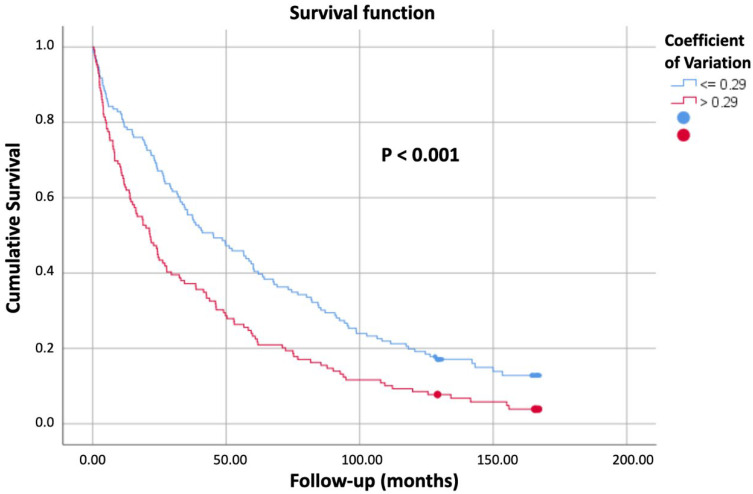
Survival curves by glucose coefficient of variation.

Table 1 summarizes the predictive factors of mortality identified in the univariate analysis. Visual inspection of the cumulative hazard function curves, stratified by MG and CV, did not demonstrate any violation of the proportionality assumption. As shown in Figure 1, patients with MG > 140 mg/dL exhibited lower cumulative survival, and similarly, Figure 2 illustrates reduced survival in those with CV > 0.29.

Figure 1 shows the Kaplan–Meier survival curves according to mean glucose levels (mg/dL). Patients with mean glucose ≤140 mg/dL showed higher cumulative survival compared with those with mean glucose >140 mg/dL (*p* = 0.006).

Figure 2 shows the Kaplan–Meier survival curves according to the glucose coefficient of variation. Patients with a coefficient of variation ≤0.29 showed higher cumulative survival compared with those with values >0.29 (*p* < 0.001).

In the multivariate analysis (Table 5), the best predictive model for mortality included, in order of prognostic importance: age (5.3% increased risk per additional year), glycemic CV during hospitalization (60% increased risk if CV > 0.29), MG during hospitalization (71% increased risk if MG > 140 mg/dL), initial eGFR (1% reduced risk per each 1 mL/min increase), history of neurological disease (46% increased risk), oxygen saturation at admission (3% reduced risk per each 1% increase), and gender (33% increased risk in males).

Table 5 shows the final multivariable Cox regression model including variables independently associated with mortality. Predictors are ordered by prognostic importance based on chi-square values. Hazard ratios (HR), 95% confidence intervals (CI), and *p*-values are provided for each variable. CV: Coefficient of Variation; GFR: Glomerular Filtration Rate.

## 4. Discussion

This study analyzed the relationship between in-hospital glycemic control—measured as MG and GV (CV)— and long-term survival after hospital discharge. In our cohort, both an MG > 140 mg/dL and a CV > 0.29 were independently and additively associated with an increased risk of mortality. The predictive power of these two variables was surpassed only by age in the best-fitting model.

In hospitalized patients, a glucose level > 140 mg/dL defines the presence of hospital hyperglycemia [13]. A recent article reviewed the recommendations of Clinical Practice Guidelines (CPG) for the management of hyperglycemia in hospitalized patients [19]. There was consensus in recommending capillary glucose monitoring during hospitalization, the use of basal and prandial insulin coverage, achieving target glucose levels between 100 and 180 mg/dL while preventing and treating hypoglycemia, and planning the patient’s transition to home care. However, no specific management protocols were provided.

The causal role of GV in mortality risk, independent of MG, remains uncertain. There is biological plausibility supporting a specific risk associated with GV [9], including increased oxidative stress and chromatin remodeling, which may contribute to the metabolic memory effect. On the other hand, there is a close relationship between GV and the risk of hypoglycemia [9], and between the risk of hypoglycemia and an increased mortality, partly due to the harmful effects of hypoglycemia itself and partly because hypoglycemia is considered a marker of frailty [20]. In the NICE-SUGAR study [21], the presence of severe hypoglycemia doubled the risk of mortality, with the effect being most pronounced (3.8-fold increase in risk) among patients who were not receiving insulin therapy. This was interpreted as an indicator of patient frailty.

Nevertheless, in the hospital setting, there are studies demonstrating the independent predictive value of GV on mortality risk both in the short- [20] and medium-term [15]. In the study by Mendez et al. [22], the 90-day mortality risk increased by 21% for every 10% rise in CV during hospitalization, although statistical significance was lost after adjusting for the presence of hypoglycemia. In the study by Akirov et al. [15], a CV > 0.29 increased the risk of mortality over a 3-year follow-up by 70%, even after adjusting for hypoglycemia.

Additional evidence from critically ill populations further supports the relevance of GV as a prognostic marker. In a recent multicenter prospective ICU study, Emgin et al. demonstrated that early GV (particularly the CV) was independently associated with 28-day mortality, reinforcing the concept that GV reflects physiological instability with meaningful prognostic implications [23].

In our study, a CV > 0.29 independently predicted mortality risk regardless of MG and the presence of hypoglycemia, although patients with higher CV also experienced a greater number of hypoglycemic episodes. Therefore, it cannot be entirely ruled out that the harmful effect of GV is partially mediated by the risk of hypoglycemia.

Spanakis et al. [24] demonstrated that the presence of hypoglycemia on the day prior to hospital discharge was associated with an increased risk of mortality at 180 days, leading them to propose careful discharge planning to improve the quality of care. Therefore, it is useful to identify patients who may benefit from more frequent follow-up after discharge, especially given the availability of care models such as day hospitals.

Currently, continuous glucose monitoring (CGM) technology allows for the assessment of GV, and CGM-derived metrics, particularly time in range (TIR), are considered complementary to HbA1c measurement [25]. The broader use of CGM may advance our understanding of the role of GV in the risk of complications and mortality in patients with diabetes [26]. The most recent CPG [19] opens the door to the future use of CGM in hospitalized patients.

The strengths of our study include the ability to conduct complete follow-up without loss in a cohort of patients with DM after hospital discharge, complementing the results of a previous study [17], and having been able to use an analysis adjusted for the principal confounding variables. Patients were treated under a standardized in-hospital management protocol [27], with frequent glucose monitoring and scheduled insulin therapy, avoiding the exclusive use of corrective insulin regimens.

The limitations of this study include the relatively small number of patients (although sufficient for the study objectives) and the inability to establish a causal relationship between glycemic measures and mortality due to the observational nature of the design, with the possibility of residual confounding. In fact, we were unable to adjust for DM duration, which could be associated with increased GV and a higher risk of mortality.

An additional aspect that should be considered is the potential confounding effect of illness severity. Hospitalized patients with more severe illness often exhibit substantially more difficult-to-regulate glycemia and greater glycemic variability, even when managed under a standardized in-hospital protocol. Therefore, illness severity may act as a confounding variable, and the association observed between hyperglycemia and mortality may be, at least in part, mediated by the adverse physiological burden of severe disease. This possibility should be taken into account when interpreting the prognostic significance of hyperglycemia in hospitalized patients.

Another important limitation is the lack of information on diabetes duration. Longer duration of diabetes has been associated with greater glycemic variability, accumulation of chronic complications, and worse clinical outcomes. The absence of this variable may therefore have introduced residual confounding, as patients with a longer disease course could be simultaneously more prone to higher GV and at increased risk of mortality. This limitation may thus have contributed to strengthening the associations observed between MG, GV, and long-term mortality.

Although data were collected during two non-consecutive periods, this characteristic of the study design did not have an impact on mortality risk, as the period effect was non-significant.

In conclusion, both MG and GV during hospitalization may serve as useful tools for stratifying mortality risk in patients after hospital discharge. Although a direct causal relationship cannot be established, the observed associations suggest that in-hospital glycemic metrics may serve as useful indicators for long-term risk stratification. In this regard, future intervention studies using CGM may help to better define the causal relationship between glycemic metrics and morbidity and mortality in patients with diabetes.

## Figures and Tables

**Table 1:** Univariate predictors of mortality.

General Variables		Total Group	Survivors	Non Survivors	*p*	HR	95% CI
Age (years)		77.6 (10.2)	67.2 (10.7)	78.7 (9.5)	<0.001	1.057	1.04–1.07
Sex (%)	Female	47.1	59.3	45.8	0.18	-	-
	Male	52.9	40.7	54.2		1.086	0.85–1.39
Stress hyperglycemia (%)		5.1	7.4	4.8	0.56	1.035	0.58–1.84
Admission for HF (%)		40.4	14.8	43.1	0.004	1.43	1.11–1.85
Neurological disease history (%)		28.6	25.9	28.9	0.74	1.49	1.13–1.96
Neoplastic disease history (%)		13.4	7.4	14.1	0.33	1.13	0.79–1.62
Charlson (points)		2.8 (1.6)	1.85 (0.91)	2.92 (1.64)	<0.001	1.12	1.04–1.20
Charlson Comorbidity index (%)	<3	47.1	77.8	43.8	<0.001	-	-
	3	25.7	18.5	26.5		1.28	0.95–1.75
	>3	27.2	3.7	29.7		1.76	1.31–2.37
SBP (mmHg)		134 (29.2)	140 (26)	137 (29)	0.59	1.001	0.99–1.01
Temperature (°C)		36.8 (0.79)	36.9 (0.77)	36.8 (0.79)	0.33	0.91	0.77–1.08
Oxygen saturation (%)		93 (5.3)	95 (3.5)	93 (5.4)	0.23	0.97	0.95–0.99
GFR (ml/min)		52.8 (24.5)	72.9 (24.2)	50.5 (23.6)	<0.001	0.989	0.98–0.99
GFR categories (%)	≥60	36.6	66.7	33.3	<0.001	-	-
	45–59	19.8	22.2	19.5		1.106	0.77–1.58
	31–44	24.9	3.7	27.2		1.79	1.29–2.48
	≤30	18.7	7.4	19.9		1.64	1.15–2.34
Sodium (mEq/L)		138 (7.5)	139 (3.8)	138 (7.8)	0.74	1.023	1.00–1.05
Hemoglobin (g/dL)		12.1 (2.8)	12.4 (2.2)	12.1 (2.8)	0.58	1.01	0.96–1.06
Leukocytes (n/mm^3^)		11,811 (7686)	10,911 (5878)	11,910 (7862)	0.52	1	1–1
Length of stay (days)		13.9 (18.4)	9.2 (4.7)	14.4 (19.3)	0.17	1.005	1–1.01
Initial glucose (mg/dL)		194 (95)	179 (101)	195 (94)	0.42	1	0.99–1.01
Initial HbA1c		7.5 (2.6)	7.7 (1.8)	7.5 (2.7)	0.59	0.99	0.93–1.05
Mean glucose on admission (mg/dL)		181 (41.7)	169 (40)	182 (42)	0.12	1.002	0.99–1.01
Mean glucose > 140 (%)		84.4	70.4	85.9	0.034	1.64	1.15–2.35
Maximum glucose on admission (mg/dL)		300 (83.4)	261 (69)	305 (84)	0.01	1.002	1.001–1.004
SD glucose		54.9 (24)	41.7 (15.9)	56.3 (24.3)	<0.001	1.009	1.004–1.014
CV glucose (0.1%)		0.3 (0.1)	0.25 (0.1)	0.31 (0.1)	<0.001	1.22	1.09–1.37
CV > 0.29 (%)		46.9	22.2	49.6	0.007	1.62	1.26–2.09
Hypoglycemia (%)	None	73.2	85.2	71.7	0.2	-	-
	Mild (≥40)	22.4	11.1	23.9		1.27	0.95–1.71
	Severe (<40)	4.4	3.7	4.5		1.48	0.80–2.72
Number of hypoglycemic episodes		0.66 (1.5)	0.19 (0.48)	0.71 (1.6)	<0.001	1.086	1.01–1.17

**Table 2:** Baseline characteristics according to the glucose coefficient of variation.

General Variables		Total Group	Coefficient of Variation ≤ 0.29 (%)	Coefficient of Variation > 0.29 (%)	*p*
Age (years)		77.6 (10.2)	76.52 (10.74)	78.66 (9.38)	0.078
Sex (%)	Female	47.1	45.9	48.8	0.62
	Male	52.9	54.1	51.2	
Stress hyperglycemia (%)		5.1	5.5	4.7	0.75
Admission for HF (%)		40.2	41.8	39.1	0.65
Neurological disease history (%)		28.6	24.7	32.6	0.147
Neoplastic disease history (%)		13.4	15.1	11.6	0.4
Charlson (points)		2.8 (1.6)	2.7 (1.6)	2.9 (1.6)	0.17
Charlson Comorbidity Index (%)	<3	47.1	49.3	45	0.08
	3	25.7	30.1	20.9	
	>3	27.2	20.5	34.1	
SBP (mmHg)		134 (29.2)	137 (28)	138 (31)	0.73
Temperature (°C)		36.8 (0.79)	36.8 (0.8)	36.8 (0.78)	0.96
Oxygen saturation (%)		93 (5.3)	93 (5.7)	94 (4.6)	0.07
GFR (ml/min)		52.8 (24.5)	54.2 (24)	51.2 (25.2)	0.33
GFR categories (%)	≥60	36.6	38.2	35.2	0.31
	45–59	19.8	19.4	20.3	
	30–44	24.9	27.8	21.1	
	<30	18.7	14.6	23.4	
Sodium (mEq/L)		138 (7.5)	138 (4.6)	138 (5.2)	0.96
Hemoglobin (g/dL)		12.1 (2.8)	12.1 (2.7)	12.2 (2.9)	0.57
Leukocytes (n/mm^3^)		11,811 (7686)	12,119 (9475)	11,460 (5019)	0.48
Length of stay (days)		13.9 (18.4)	13.4 (21.3)	14.3 (14.7)	0.71
Mean glucose on admission (mg/dL)		181 (41.7)	176 (46.3)	186 (35.4)	0.064
Mean glucose > 140 (%)		84.4	78.1	91.5	0.002
Maximum glucose on admission (mg/dL)		300 (83.4)	261 (69.6)	346 (74.1)	<0.001
Hypoglycemia (%)	None	73.2	92.4	50.8	<0.001
	Mild (≥40)	22.4	7.6	39.8	
	Severe (<40)	4.4	0	9.4	
Number of hypoglycemic episodes		0.66 (1.5)	0.10 (0.38)	1.30 (2)	<0.001

**Table 3:** Baseline characteristics according to mean glucose levels.

General Variables		Total Group	Mean Glucose ≤ 140	Mean Glucose > 140	*p*
Age (years)		77.6 (10.2)	77.2 (10.04)	77.64 (10.22)	0.79
Sex (%)	Female	47.1	41.9	48.1	0.45
	Male	52.9	58.1	51.9	
Stress hyperglycemia (%)		5.1	4.7	5.2	0.89
Admission for HF (%)		40.4	37.2	40.9	0.65
Neurological disease history (%)		28.6	20.9	30	0.22
Neoplastic disease history (%)		13.4	16.3	12.9	0.55
Charlson (points)		2.8 (1.6)	2.28 (1.14)	2.91 (1.67)	0.017
Charlson Comorbidity Index (%)	<3	47.1	60.5	44.6	0.024
	3	25.7	25.6	25.8	
	>3	27.2	14	29.5	
SBP (mmHg)		134 (29.2)	133 (28.2)	139 (29.3)	0.024
Temperature (°C)		36.8 (0.79)	36.8 (0.72)	36.8 (0.8)	0.72
Oxygen saturation (%)		93 (5.3)	94 (6)	93 (5.1)	0.43
GFR (ml/min)		52.8 (24.5)	51.8 (21)	52.9 (25.1)	0.78
GFR categories (%)	≥60	36.6	32.5	37.3	0.85
	45–59	19.8	27.5	18.5	
	30–44	24.9	25	24.9	
	<30	18.7	15	19.3	
Sodium (mEq/L)		138 (7.5)	139 (4.6)	138 (7.9)	0.80
Hemoglobin (g/dL)		12.1 (2.8)	11.5 (2.8)	12.2 (2.8)	0.12
Leukocytes (n/mm^3^)		11,811 (7686)	10,802 (5546)	11,990 (8001)	0.36
Length of stay (days)		13.9 (18.4)	14.4 (24.2)	13.8 (17.2)	0.84
SD glucose		54.9 (24)	29.4 (12)	59.6 (22.7)	<0.001
CV glucose		0.3 (0.1)	0.23 (0.1)	0.31 (0.1)	<0.001
CV > 0.29 (%)		46.9	25.6	50.9	0.002
Hypoglycemia (%)	None	73	82.9	71.2	0.13
	Mild (≥40)	22.6	14.6	24	
	Severe (<40)	4.4	2.4	4.7	
Number of hypoglycemic episodes		0.66 (1.5)	0.44 (1.2)	0.69 (1.6)	0.32

**Table 4:** Mortality rates per 1000 patient years.

	Patients	Follow-Up (Years)	Patients x Follow-Up	Deaths	Rate/1000
All	276	4.26	1175.76	249	211.7779139
Male	144	4.21	606.24	135	222.6840855
Female	128	4.39	561.92	114	202.8758542
Glucose ≤ 140	40	6.37	254.8	35	137.3626374
Glucose > 140	232	3.94	914.08	214	234.1151759
CV ≤ 0.29	144	5.16	743.04	125	168.2278208
CV > 0.29	128	3.33	426.24	123	288.5698198
No neurological disease	195	4.79	934.05	177	189.4973502
Neurological disease	77	3.05	234.85	72	306.5786672
CCI < 3	130	4.99	648.7	109	168.0283644
CCI = 3	71	4.12	292.52	66	225.6255982
CCI > 3	71	3.19	226.49	74	326.7252417
GFR ≥ 60	100	5.01	501	82	163.6726547
GFR 45–59	54	4.98	268.92	48	178.4917448
GFR 30–44	67	3.22	215.74	67	310.5590062
GFR < 30	51	3.43	174.93	49	280.1120448

**Table 5:** Multivariable Cox regression analysis with variables ordered by prognostic importance.

General Variables	X^2^	HR	IC 95%	*p*
Age (years)	39.758	1.053	1.036–1.071	<0.001
CV > 0.29	12.037	1.600	1.228–2.085	0.001
Mean blood glucose > 140 mg/dL	8.393	1.708	1.164–2.506	0.004
Initial GFR (ml/min/1.73 m^2^)	6.362	0.993	0.987–0.998	0.012
Neurological disease history (%)	6.274	1.462	1.094–1.954	0.012
Oxygen saturation at admission (%)	6.124	0.969	0.946–0.992	0.013
Sex (male)	4.448	1.331	1.020–1.736	0.035

## Data Availability

The data presented in this study are not publicly available due to ethical and privacy restrictions related to patient clinical information. Data may be available from the corresponding author upon reasonable request and with permission from the Ethics Committee.

## Abbreviations

ANOVA: Analysis of Variance; CGM: Continuous Glucose Monitoring; CCI: Charleston Comorbidity Index; CI: Confidence Interval; CKD-EPI: Chronic Kidney Disease Epidemiology Collaboration; CPG: Clinical Practice Guidelines; CV: Coefficient of Variation; DM: Diabetes Mellitus; eFGR: Estimated Glomerular Filtration Rate; GFR: Glomerular Filtration Rate; GV: Glycemic Variability; HbA1c: Hemoglobin A1c; HF: Heart Failure; HR: Hazard Ratio; ICU: Intensive Care Unit; KDIGO: Kidney Disease Improving Global Outcomes; MG: Mean Glucose; R: Software R; SBP: Systolic Blood Pressure; SD: Standard Deviation; SPSS: Statistical Package for the Social Sciences; TIR: Time In Range.
